# The chloroplast genome of the pincushion cactus *Mammilllaria haageana* subsp. *san-angelensis*, a Mexican endangered species

**DOI:** 10.1080/23802359.2020.1757523

**Published:** 2020-05-12

**Authors:** Silvia Hinojosa-Alvarez, Salvador Arias, Sylvie Ferrand, Michael D. Purugganan, Julio Rozas, Ulises Rosas, Ana Wegier

**Affiliations:** a Jardín Botánico, Instituto de Biología, Universidad Nacional Autónoma de México, Coyoacán, México City, México; b Center for Genomics and Systems Biology, New York University Abu Dhabi Research Institute, Abu Dhabi, United Arab Emirates; c Center for Genomics and Systems Biology, New York University, New York, NY, USA; d Departament de Genètica, Microbiologia i Estadística, Universitat de Barcelona (UB) and Institut de Recerca de la Biodiversitat (IRBio), Barcelona, Spain

**Keywords:** Cactaceae, phylogenomics, rare species, conservation genetics, succulent plants

## Abstract

The genus *Mammillaria* occupies diverse habitats and exhibits diverse growth patterns and a large range of morphologies. Most of the species of this genus are used as ornamental plants and are subject to mass habitat loss. Due to these factors, they are being submitted to selective pressure that might affect conservational efforts and management plans. We obtained the 133 gene chloroplast genome as part of the project of sequencing the complete genome of pincushion cactus, including 88 protein-coding genes, 8 rRNA genes, and 37 tRNA genes. The phylogenetic tree indicates the pincushion cactus is a sister species of *M. supertexta* and *M. huitzilopochtli*.

The *Mammillaria* genus is comprised of small succulent plants, which are highly diverse in growth habits and morphologies, making it the most species-rich genus within the Cactaceae family and among the top five species-rich genus in Mexican vascular plants (Villaseñor 2016). Among its 166 species distributed in the Americas, 150 species are endemic to Mexico, with many species having narrow distributions (Hernandez and Godinez 1994). This species requires more comprehensive and robust conservation strategies than widely distributed taxa; like the pincushion cactus (*Mammillaria haageana* subsp. *san-angelensis*), whose distribution is restricted to the Ecological Reserve of Pedregal de San Ángel (REPSA) in Mexico City (Arias 2009; Valverde and Chávez 2009). This makes the species an ideal model to understand ecological and evolutionary implications. Here we present the chloroplast genome of *M. haageana* subsp. *san-angelensis* as an initial approach toward developing genomic tools to better understand *M. haageana* and its close relatives.

We sampled an individual of the pincushion cactus (JardínBotánico/UNAM = C-IC-02-03) from the live collection of the University Botanical Garden (19.3206° N, 99.1944° W). Total genomic DNA was extracted using DNeasy Plant Mini Kit (Qiagen, Hilden, Germany) following the manufacturer’s instructions. High-throughput DNA sequencing was conducted using the *NovaSeq6000* System with S1 FlowCell (Illumina Inc., San Diego, CA) 2 × 150 bp. After base quality control using Trimmomatic v0.32 (Bolger et al. 2014), the remaining high-quality reads were used to assemble the chloroplast genome by Novoplasty 3.7.1 (Dierckxsens et al., 2016) using *M. supertexta* chloroplast genome reference (Solórzano et al. 2019) and *rpl16* gene (Genbank accession number: AY545323.1) as seed, following the software specifications (Dierckxsens et al., 2016). The plastome was annotated using GeSeq (Tillich et al., 2017). The accurate gene boundaries were confirmed by alignment with other chloroplast genes of Cactaceae using MAFFT v7.311 (Katoh et al., 2019). We generated a maximum-likelihood (ML) tree through IQTREE (Nguyen et al. 2015) with 1000 bootstrap alignments and SH-aLRT and Bayes tests with 1000 replicates based on 11 complete chloroplast sequences aligned with *Dionaea muscipula* as outgroup.

The complete chloroplast genome of *M. haageana* subsp. *sanangelensis* was determined to be 115,386 bp in length, similar to those previously reported by Solórzano et al. 2019 for another *Mammillaria* species. The *M. haageana subsp. san-angelensis* genome has 133 genes, including 88 protein-coding genes, 8 rRNA genes, and 37 tRNA genes.

The ML analysis reveals that *M. haageana* subsp. *san-angelensis* is a sister to *M. supertexta* forming monophyletic group closely related to *M. crucigera* and *M. huitzilopochtl*i (Figure 1).

**Figure 1.**
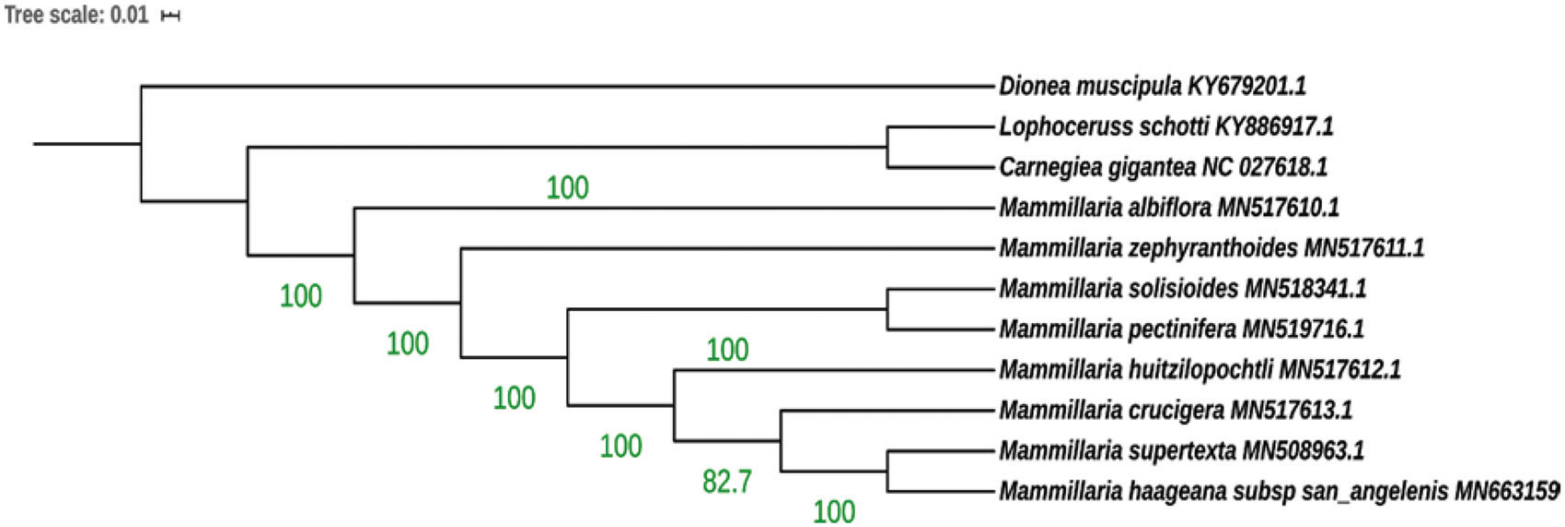
Molecular phylogeny of *M. haageana* subsp. *san-angelensis* constructed by Maximum likelihood method. *Mammilllaria haageana* subsp. *san-angelensis* has been considered a subspecies for more than 30 years, but our initial results may indicate that it could be elevated to species status. Further studies including complete chloroplast phylogenomics and morphology will be useful to elucidate the *Mammillaria* phylogenomics.
